# Bioinspired Designs for Lightweighting, a Critical Review for Manufacturing

**DOI:** 10.3390/biomimetics10030150

**Published:** 2025-03-01

**Authors:** Vinay Kenny, Salil Bapat, Pauline Smith, John La Scala, Ajay P. Malshe

**Affiliations:** 1 Manufacturing and Materials Research Laboratory (MMRL), School of Mechanical Engineering, Purdue University, West Lafayette, IN 47906, USA; 2 DEVCOM Army Research Laboratory, Aberdeen Proving Grounds, Aberdeen, MD 21005, USA

**Keywords:** bio-inspired, lightweight, design, manufacturing

## Abstract

The design and manufacturing of lightweight structures (also termed lightweighting) are essential for many industrial applications to reduce material and energy consumption, impacting industries from automobiles to aerospace. Through millions of years of evolution, biology has utilized intricate designs and materials that are both lightweight and strong as a part of evolution, enabling organisms to adapt efficiently to their environments and providing a library of lightweighting approaches. This paper provides a comprehensive overview of biological design strategies for lightweighting. The authors introduce a biological design toolbox for lightweighting, a modular list of design attributes biological species utilize to develop lightweight structures. Selected representative lightweight biological examples and the fundamental science governing their design strategies are analyzed and discussed using the design toolbox, which could be applied in manufacturing engineered parts and systems. Their corresponding simulated and/or manufactured designs were also studied to highlight the gaps and opportunity space in the current bio-inspired design practices. To address these gaps, a holistic bio-inspired design framework for lightweighting is proposed as a part of future research based on the critical analysis of the design toolbox for lightweighting.

## 1. Introduction

In today’s increasingly natural resource-constrained environment, many industrial sectors continue the pursuit of enhancing efficiency, sustainability, and performance by emphasizing lightweighting as one of the key strategies [1]. Lightweighting, at its core, is the strategic reduction in the weight of the products without compromising their structural integrity, performance, and functionality. As an example of an application area in automotive manufacturing, stringent regulatory standards aimed at reducing carbon footprint and enhancing energy efficiency have intensified the focus on lightweighting strategies [2,3]. Similarly, in aerospace, where all additional weight directly impacts fuel consumption and operational costs, lightweighting is prominent in improving aircraft efficiency and range [4,5]. Typical strategies for reducing weight include the incorporation of porous designs as well as topology optimization, which is very common for additive manufacturing-enabled customizations [6,7,8]. However, achieving a balance between efficiency and effectiveness as well as achieving weight reduction without compromising structural integrity and performance and advancing the desired functionality remains a persistent challenge.

**Figure 1:**
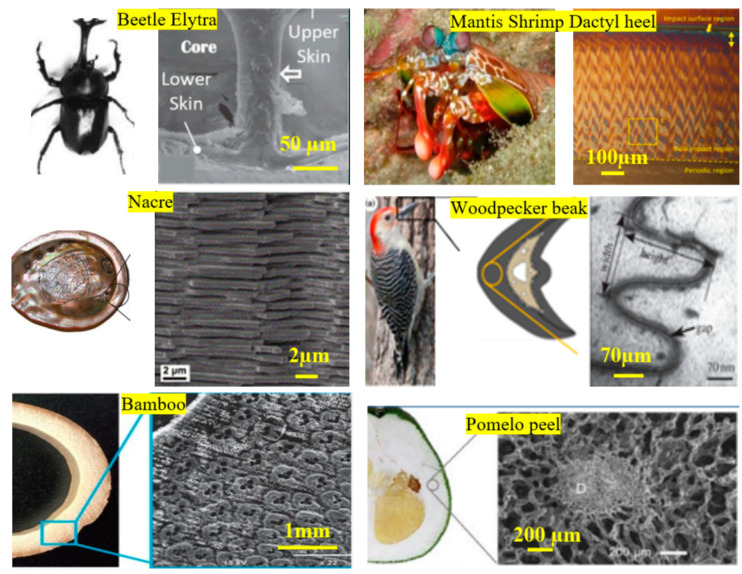
Examples of lightweight bio parts with energy absorption capability and design features. Images reproduced with permission from Refs. [12,13,14,15,16,17,18,19].

Over billions of years, through evolution, biology has facilitated the growth and survival of diverse biological species on Earth. Evolution entails selecting the most effective design strategies addressing the challenges faced by species, thereby fostering adaptation and survival [9]. Biology has exemplified ingenious methods to enhance design effectiveness while maximizing functionality within the constraints of its limited material resources. The principle of “minimizing material use while maximizing performance” is crucial for creatures to endure harsh environments [10]. Consequently, design attributes observed in biological species have been applied to design several functional products [11]. Figure 1 shows representative examples of the design architectures for lightweighting utilized by various species from diverse environments specifically for achieving impact resistance. These designs are influenced by environmental interactions and material constraints, leading to the distinct organization of different design elements, resulting in advanced mechanical properties.

This paper is focused on understanding the role of various design architectures found in biology to achieve lightweighting, viewed through the lens of biological design strategies and underlying scientific principles. Towards this, the following section is focused on the discussion of the biological design toolbox for functional lightweighting. Various representative biological examples for lightweighting, detailing their design strategies, working mechanisms, and underlying scientific principles to achieve a desired functional response, are presented in Section 3. Representative case studies of various manufactured and/or simulated bioinspired lightweight designs are discussed in Section 4 to illustrate current approaches to designing and manufacturing bio-inspired lightweight structures. The paper concludes with a summary and future directions, highlighting the gaps and opportunities in the traditional bioinspired design methodology while proposing a holistic design framework for manufacturing bio-inspired lightweight architectures.

## 2. Biological Design Toolbox (BDT) for Functional Lightweighting

**Figure 2:**
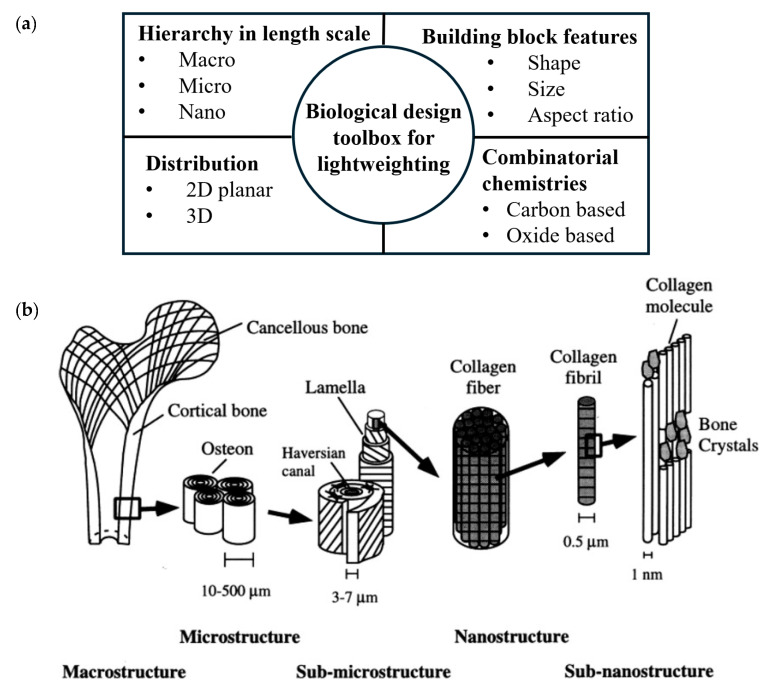
(**a**) Biological design toolbox (BDT) for functional lightweighting: a modular toolset in biology, (**b**) pictorial representation for the hierarchical structural design of human bone. Reproduced with permission from Ref. [23].

Biology offers many examples of lightweight structures, showing a vast library of designs optimized for functionality and efficiency. Studying and analyzing these designs through various scholarly publications reveals a systematic ensemble of tools utilized in biological designs, presented here as a biological design toolbox (BDT; Figure 2a). It should be noted that the design toolbox in Figure 2a schematically depicts only the primary design attributes with representative examples for illustration. To illustrate the toolbox’s taxonomy, human bone design is used as a reference (Figure 2b), as it exemplifies a balance between strength, lightweight structure, and biological functionality [20,21]. Despite cortical bone being dense and relatively heavy, its high specific strength makes it comparatively lightweight [22].

Below are the insights and discussion of the design parameters shown in the toolbox (Figure 2a), along with a follow-up brief description using bone structure (Figure 2b) as a representative example.

1. Combinatorial chemistries: The design parameter chemistry refers to the material and/or combination of materials chemistries used to construct the design. The material composition of the design structure plays a crucial role in determining its mechanical and functional properties. Example: Collagen fibers comprise collagen, a structural protein in connective tissues throughout the body. This protein predominantly includes amino acids, glycine, proline, and hydroxyproline [24]. These amino acids form a triple helix structure, giving collagen strength and flexibility [25].
2. Features: A feature is defined as a unit three-dimensional (D) building block (BB) of a structure. The BB has three key attributes that define the 3-D design parameter: size, shape, and aspect ratio. Example: The fibrous BB in bone, primarily collagen fibril, is vital in providing tensile strength and flexibility [23]. The BB can be described as a cylindrical shape, size characterized by its diameter and wall thickness, and the ratio of diameter to height defines its aspect ratio. Depending on the biological structure, the shapes can become more complex.
3. Distribution: This design parameter considers the arrangement of features in a 2-D and 3-D physical space. Example: In bone structure, collagen fibrils form strands known as collagen fibers, which exhibit a 2-D radial arrangement. These strands are then oriented according to the required strength in a specific direction, creating lamellae. The 2D/3D distribution can be subdivided into directionality, periodicity (aperiodicity), gradient, and order (disorder) of arrangements and their mutual combinations (or lack thereof).
  - Directionality refers to the features’ assembly and orientation in the 2-D or 3-D space, resulting in isotropic or anisotropic properties.
  - Periodic arrangement of features is a repeating or constant pitch of features or material. The periodicity can be observed along and across multiple levels.
  - A gradient is the variation in a structure’s density or distribution of material or feature.
  - An order refers to the positioning or arrangement of features in a specific sequence within a structure. Although random, the randomness is mostly by design, which involves a strategic arrangement of features based on location-specific functional requirements.
4. Hierarchy: Hierarchy refers to the organization of features or clusters of features in the system at different length scales (from nano-to-micro-to-macro) and/or along specific directions (e.g., axial direction or radial direction). Example: Bone’s hierarchical structure (as depicted in Figure 2b: from nanoscale collagen to microscale lamella and osteons to macroscale bones) has an irregular but optimized arrangement and orientation of its components, resulting in heterogeneous and anisotropic bone material [26].

The combinatorial organization of the above-discussed design parameters in biology achieves various lightweight, strength-to-weight ratios and other multi-functional design schemes. These design parameters depicted in Figure 2a can be independently defined for a biological structure. However, there is often overlap and interaction between these design parameters, as changes in one parameter can influence or be influenced by others. This interdependency contributes to the overall multi-functionality for effectiveness and efficiency of the biological structure(s), as discussed below through representative biological examples. The examples discussed below were chosen by filtering papers from the scientific literature using ‘bio-inspired or biological’ and ‘lightweight designs or lightweight structures’ as keywords (and combinations thereof), which specifically discuss the design geometry of various biological species and/or discuss the underlying scientific mechanisms.

## 3. Biological Examples and Fundamental Science for Achieving Lightweighting

This section discusses representative examples of various biological design strategies for lightweighting and their underlying scientific principles while maintaining strength and desired functionality. The anatomy and the structural designs of multiple species in diverse environments are examined and analyzed for the specific functionality these lightweight designs deliver. Based on this information, the parameters responsible for functional lightweighting are identified for each species as an example case study. These identified parameters are then connected to BDT at the end of each subsection as a part of the subsection summary and correlation with other sections.

### 3.1. Lightweight Biological Examples with High Stiffness

Stiffness is crucial for maintaining dimensional stability and preventing deformation under load. This property is especially evident and necessary in plant species that grow upright. Due to their inherent slenderness, these plants are susceptible to forces from their own weight and wind conditions. This susceptibility can cause permanent deformation and severely impact their ability to survive.

Bamboo is an example of a plant well known for its ability to withstand significant wind loads with negligible deformation due to its exceptional directional stiffness. It is one of the fastest-growing plants in the world, with a peak growth rate of 100 cm per day [27]. Despite having large aspect ratios and being hollow, it possesses a stiffness-to-mass ratio exceeding that of metallic materials like steel [15,28].

**Figure 3:**
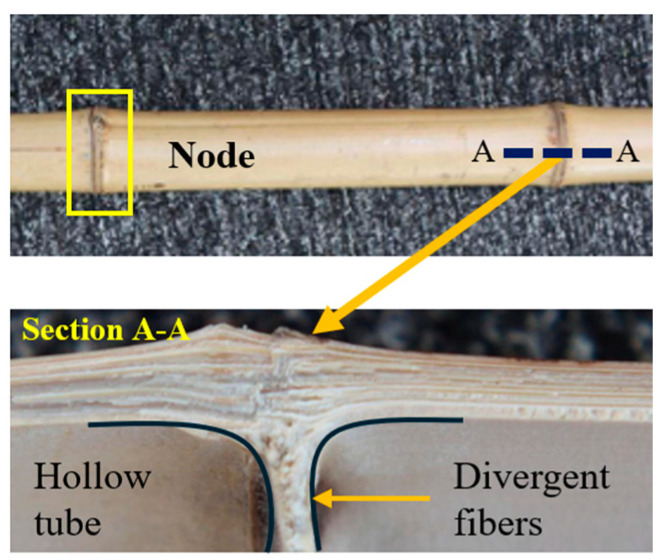
Bamboo’s exterior and cross-sectional images showing nodes and fiber orientations. Reproduced with permission from Ref. [33].

Bamboo consists of hollow tubes and solid segmented nodes at the macro-scale, as seen in Figure 3. The highly oriented fibers diverge from the longitudinal path around nodes, so the overall structure is highly anisotropic. The tubular design is essential to achieve the required stiffness. The relationship between this design and the resulting stiffness can be explained using beam curvature and Euler’s buckling equations, which help analyze the effect of applied load on the deformation. These equations govern the fundamental mechanics of elastic deflections and are related to the geometrical characteristics of the structure.

The relationship between the bending moment, *M*, and the curvature of the beam is described by the equation below [29]:

(1) $$\frac{\partial^{2}y}{\partial x^{2}}=\frac{M}{EI}$$

where y is the beam’s deflection in the direction x, *E* is the modulus of elasticity, and *I* is the cross-section’s moment of inertia. For a solid cylinder of radius *R*, the moment of inertia is calculated as per Equation (2) [30].

(2) $$I_{cylinder}=\frac{\pi R^{4}}{4}.$$

Thus, the curvature of the beam, which essentially dictates its deflection, is inversely proportional to *I* or the fourth power of the radius [31]. Therefore, the deflection can be reduced by increasing *I*, which implies increasing the radius. Euler’s buckling equation is used to calculate the compressive load at which global buckling takes place and is described as [32]:

(3) $$P_{cr}=\frac{\pi^{2}EI}{{\left(kL\right)}^{2}},$$

where *k* is the constant that depends on the fixturing conditions, and *L* is column length. Similar to the beam curvature, resistance to buckling can be enhanced by increasing the radius, thereby increasing *I.* Equations (1) and (3) represent the governing design boundary conditions for lightweight and/or stiff structures [31].

For two cylindrical bodies with equal masses, *I* can be increased by positioning the mass farthest from the neutral axis [32]. This can be achieved by generating a hollow tube with a radius R and thickness t [31]. The ratio of the moment of inertia of a hollow cylinder (tube) to a solid cylinder of the same mass is given by

(4) $$\frac{I_{tube}}{I_{cylinder}}=1+x^{2};x=1-\frac{t}{R}.$$

Thus, to increase the resistance to bending, t should be minimized, and *R* should be maximized. Local buckling decreases with a decrease in t and an increase in *R* [31]. With the example of bamboo, the resistance against bending and buckling has been addressed by creating a thin-walled tubular structure.

**Figure 4:**
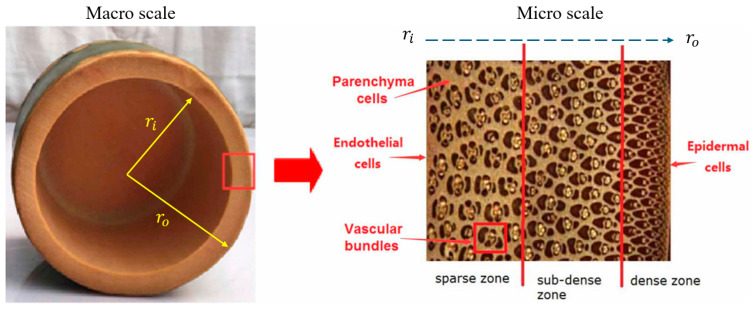
Structural design of bamboo plant detailing its macro- and micro-scale features. Reproduced with permission from Ref. [34].

At the microscopic level, bamboo exhibits a hierarchical tubular design with vascular bundles arranged with gradient density, as depicted in Figure 4. Vascular bundles are essential structural building blocks in the bamboo design, providing mechanical support and facilitating water and nutrient transport. These bundles are organized with radially increasing density from the inner surface to the outer surface. This configuration optimizes material usage by positioning the most challenging design configuration in the location of high-stress regions (towards the outer edge) [34]. Bamboo’s structural design consists of cellulose microfibrils and lignin constituents. The hollow cavity design and gradient density distribution with different porosity levels results in a lightweight design while delivering the desired stiffness due to the plant’s tubular geometry.

The radial gradient density of strong vascular bundles and the bamboo’s tubular structure further add to the resistance to bending, explained using the theory of bending stress. The fundamental mechanic’s equation relating bending stress at a radial distance y from the centroid is expressed as:

(5) $$\sigma_{y}=\frac{My}{I}.$$

Due to the linear increase in stresses with y, the central core experiences minimal stress and does not contribute significantly to flexural resistance. This provides a rationale for using a hollow core in bamboo where the material with greater strength is positioned towards the outer portion of the radial structure.

**Figure 5:**
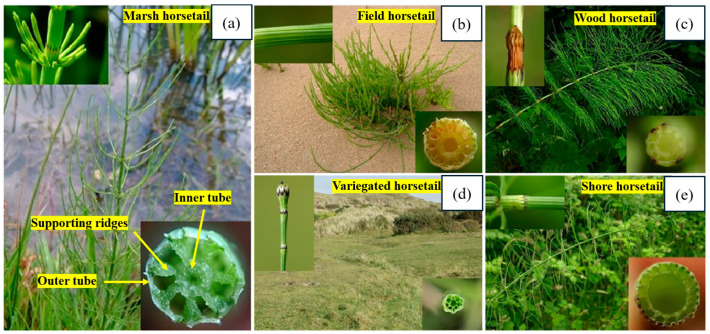
Designs of various horsetail plants showing inner tubes, outer tubes, and supporting ridges, (**a**) marsh horsetail, (**b**) field horsetail, (**c**) wood horsetail, (**d**) variegated horsetail, and (**e**) shore horsetail. Reproduced with permission from Ref. [35].

**Figure 6:**
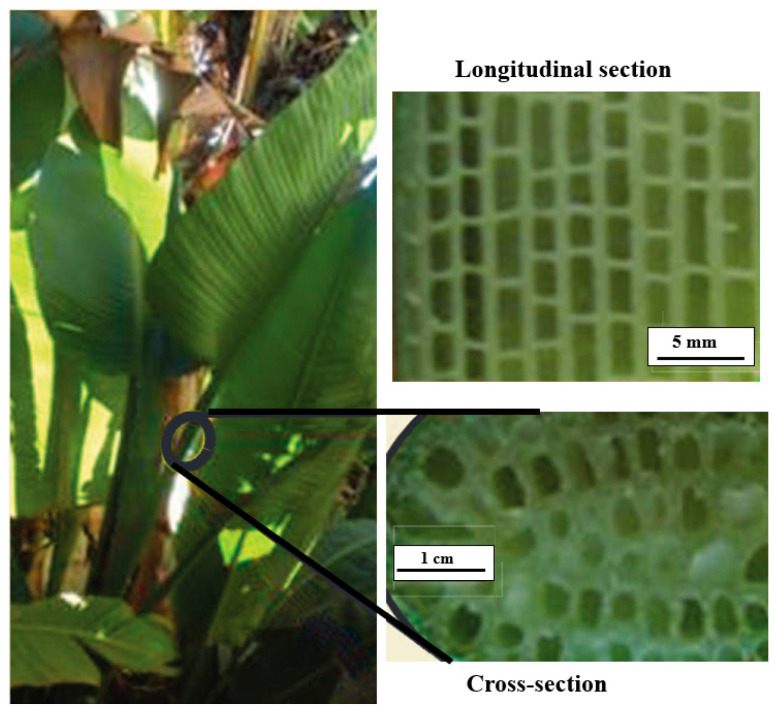
The multi-celled tubular design of the stem of a giant bird of paradise plant. Reproduced with permission from Ref. [31].

Like bamboo, the bird of paradise and horsetail plants also demonstrate specific stiffness properties using their unique cellular designs. Different varieties of horsetail plants feature a concentric tubular design supported by internal ridges, as depicted in Figure 5. The primary structural feature contributing to its stiffness is the multi-celled tubular arrangement [35]. On the other hand, the ‘bird of paradise’ plant features radially aligned tubular cells, as depicted in Figure 6. In the longitudinal section, the cells have a rectangular appearance. In contrast, their walls are radially aligned in the cross-section, creating a hollow cylindrical shape. The struts incorporate a pattern of holes, creating a porous structure that helps significantly reduce weight. Similar to bamboo, the structural designs of these plants are engineered to withstand flexural stresses without buckling [31].

Table 1 summarizes how the key design parameters from the lightweighting toolbox were utilized to realize lightweight structures while enabling high stiffness. The commonalities include the hierarchical distribution of features from micro to macro scale, the presence of fibrous structures, and porous cells. It also highlights how the various design parameters from Figure 2a interact to collectively achieve a desired functionality (stiffness, in this case). The three examples discussed above also demonstrate different permutations of this interaction, resulting in the usage of various materials and other arrangements of features, possibly to account for different operational environments and boundary conditions. As such, selecting specific biological designs could be considered an optimization of various design parameters under the environment/habitat-imposed boundary conditions and resource constraints to achieve effective functional performance from a systems-level perspective. This approach is foundational to the theme of this paper and is explored in further detail in Section 5.

### 3.2. Lightweight Biological Examples with Impact Resistance

Some biological species, such as mantis shrimp, beetles, and nacre, have evolved to combine lightweight structures with remarkable impact resistance. These functionalities enhance their agility during attack and provide robust defense mechanisms against environmental impacts and predators. At the core of this is toughness, which is the ability of a material to absorb energy and resist breaking or cracking when subjected to stress or impact.

**Figure 7:**
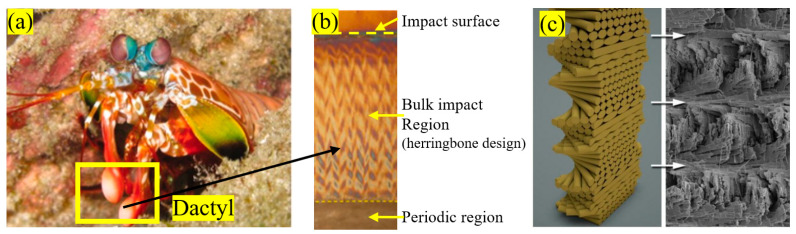
Design of peacock mantis shrimp: (**a**) shrimp’s dactyl, (**b**) different dactyl regions, (**c**) dactyl herringbone design generated from a scanning electron microscopy image of a fractured surface. Reproduced with permission from Ref. [38].

The punch of a peacock mantis shrimp can produce an acceleration exceeding 105 m/s^2^ and reach an impact speed of over 20 m/s, making it one of the most potent and fastest impact events seen in nature [10,36] Mantis shrimp can endure impact forces of up to 1500 N without catastrophic damage [37]. This discussion will focus on two biological parts of mantis shrimp: the dactyl heel and the telson. The dactyl heel of mantis shrimp is a specialized appendage on its limb, specifically, the segment closest to the body, as seen in Figure 7, used primarily for attacking the prey. The dactyl heel of the mantis shrimp exhibits a highly ordered and repeating herringbone pattern (Figure 7c), which consists of chitin fibrils stacked upon each other at staggered angles. The energy dissipation mechanisms in this impact region involve crack deflection and twisting. The spiraled structure of the shrimp’s dactyl heel absorbs impact similar to a spring, redirecting any cracks that may form within the material, thereby reducing their force and resisting direct crack propagation. In this case, the chemical composition of the material and the interfaces play a vital role in achieving the desired mechanical properties. When stressed, the mineral components, for example, calcite, fracture, whereas the chitin fibers can absorb the strain [10].

**Figure 8:**
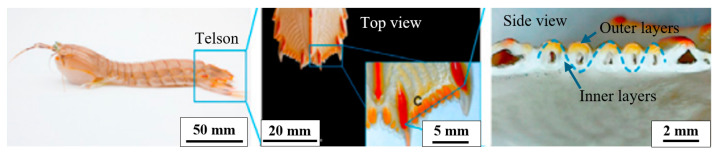
The design of the shrimp’s telson depicting the wavy arrangement of hard and soft layered features. Reproduced with permission from Ref. [41].

Developing impact-resistant armor, the telson, is crucial for the shrimp to endure forceful predator strikes. The design of the telson is represented in Figure 8. The primary reason for the telson’s impact resistance is the combination of its hard and stiff outer layers, which prevent penetration during impact, and its flexible inner layers, which contribute to toughness [39]. This increase in the toughness is attributed to crack deflection caused by the local changes in stiffness and strength, delaying catastrophic failure. Telson typically exhibits higher spring stiffness, reduced deformation, and shorter contact durations, resulting in increased impact forces and a greater likelihood of potential damage from the impact. As a secondary mechanism, the telson’s coil position enhances its ability to flex and displace significantly, helping to reduce impact forces. The telson’s design function is similar to a relatively stiff spring, effectively dissipating most impact energy [40].

**Figure 9:**
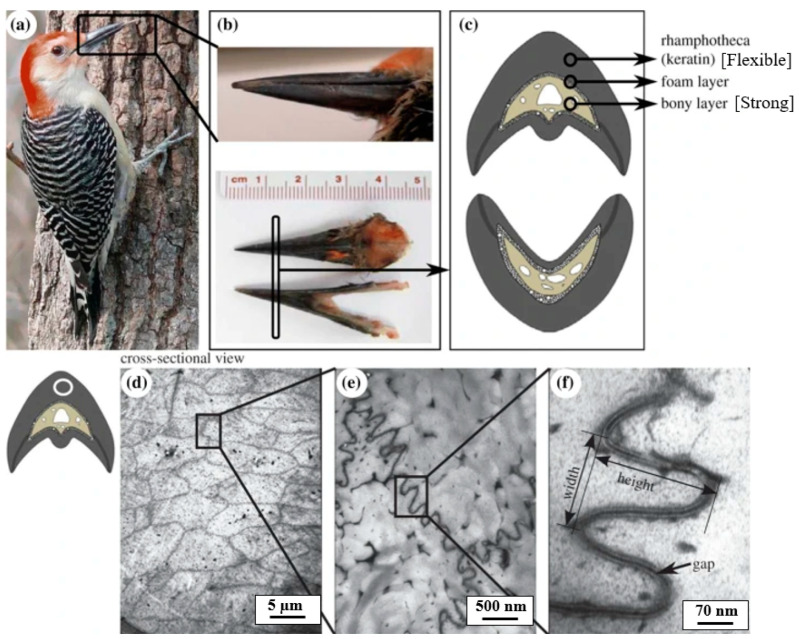
Design of the woodpecker’s beak: (**a**) woodpecker, (**b**) the beak of the woodpecker, (**c**) strong and flexible beak region, (**d**) micrograph of the flexible regions, (**e**) wavy honeycomb structure, and (**f**) magnified view of the wavy region. Reproduced with permission from Ref. [15].

**Figure 10:**
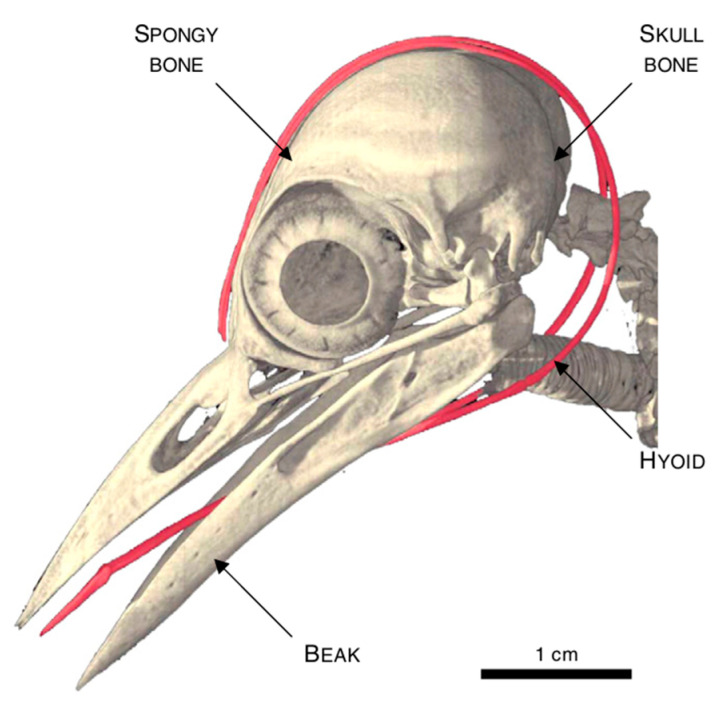
Design of woodpecker’s beak and hyoid region responsible for energy dissipation [44].

Woodpeckers represent another example of impact-resistant biological structures. Woodpecker beaks repeatedly strike with a force of 1 kgf at speeds of 6–7 m/s without sustaining head injuries [42]. When a woodpecker’s beak hits an object, the intense impact force at the tip is mitigated by the structure of the beak (Figure 9) and the spongy geometry of the hyoid bone, which absorbs shock and prevents head injuries. The energy dissipation of impact forces in the woodpecker occurs in various stages. The first point of contact is the beak, which has a strong, dense interior layer of bone and a flexible exterior layer of tissue, as shown in Figure 9c. This exterior layer absorbs initial impact forces, using its flexibility to withstand stress and minimize shock by bending and flexing during vibrational transmission. The beak connects with a delicate system of bone, tendon, and muscle surrounding the skull, known as the hyoid, and forms a dissipation network, as shown in Figure 10. The forces then travel along the hyoid, bypassing the skull and exiting through the tongue [43]. In this complex energy dissipation mechanism involving different parts of the woodpecker, the authors focus on the nano-scale design of the beak. Figure 9f shows the nanostructure of a woodpecker’s beak, revealing its tightly packed and wavy configuration with narrow gaps along the edges [10]. This jigsaw-like structure optimizes energy absorption by enabling local shearing and combining low friction coefficients between surfaces with high interlocking angles [15].

**Figure 11:**
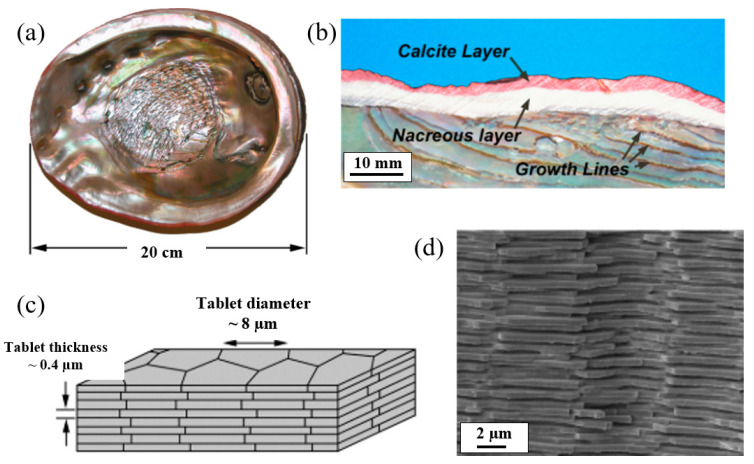
Design of nacre: (**a**) abalone shell, (**b**) the material arrangement, (**c**) tablet’s schematic, (**d**) SEM image of a fracture surface. Reproduced with permission from Ref. [47].

Nacre’s shell demonstrates impressive impact resistance despite its inherent brittleness owing to its unique design configuration [45]. The shell consists of outer and inner layers made up of large prismatic calcite grains (calcite layer) and nacre (nacreous layer) at the macro-scale, forming an efficient armor system, as shown in Figure 11b. The hard calcite layer resists external penetration, while the softer nacreous layer dissipates impact energy, preventing catastrophic failure. At the microscale, mineral biological tablets, depicted in Figure 11c,d, are stacked in organized columns with adhesive biopolymers at their interfaces, aiding energy absorption [10]. The design strategy employs a three-dimensional brick-and-mortar arrangement of plates and manages the hard–soft material interfaces [46], enabling effective energy absorption despite the inherent brittle nature of the calcite tablets.

The fundamental mechanical property governing the impact resistance is toughness, which quantifies the amount of energy a material can absorb before failure, typically expressed as [31]:

(6) $$U=\int_{0}^{\epsilon_{f}}\sigma \;d\epsilon ,\;$$

where U is the energy per volume absorbed, σ is stress, ϵ is strain, and $\epsilon_{f}$ is the failure strain. Typically, tougher materials exhibit significant plastic deformation, which results in an increased integral value. For lightweighting applications, the parameter of interest is usually the specific energy absorption (SEA), which is the absorbed energy per unit mass (m) of the structure and is given by [17]:

(7) $$SEA=\frac{U}{m}.$$

The toughening mechanisms observed in the nacre’s shell, the woodpecker’s beak, and the mantis shrimp’s dactyl heel discussed above depend on carefully architectured multi-material interfaces. When a crack encounters an interface or discontinuity within the material, it can deflect around the interface or propagate through it. In both cases, additional energy is required for crack propagation compared to a similar structure without any crack-arresting interface [31]. Thus, interfaces and associated discontinuities enhance the structure’s energy absorption capability. On the other hand, some design-related features, as demonstrated in the case of shrimp’s telson, contribute to increased energy absorption.

The key design parameters from the BDT responsible for delivering the impact resistance in biological lightweight structures are summarized in Table 2. A combination of hard and soft materials, hierarchical and periodic arrangements of features, crack-arresting interfaces, and directional arrangement of features can be viewed as primary strategies to deliver high impact resistance to these structures.

### 3.3. Lightweight Biological Example with Torsional and Bending Resistance

**Figure 12:**
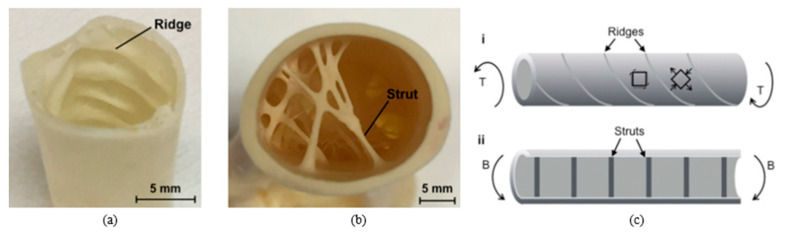
The structure of avian wing bone showing (**a**) ridges, (**b**) struts, and (**c**) schematic representation of (**i**). ridges, and (**ii**). struts. Reproduced with permission from Ref. [50].

Flighted birds are an exceptional example of species demonstrating torsional and bending resistance while being lightweight. Birds need to minimize their weight to minimize flight costs, but at the same time, skeletons need to be strong enough to withstand the aerodynamic forces during flight. Many bird bones are composed primarily of cortical bone, which is less porous than any other type of bone, making it a dense tissue [48]. Skeletons of birds can be classified as lightweight structures with respect to strength per unit of mass. The wing skeleton is exceptionally lightweight; in contrast to the marrow-filled bones of terrestrial vertebrates, most bird wings consist of hollow bones [49]. A thin, dense exterior with a hollow interior provides resistance to bending and torsional moments. The equation governing the torsional stress, τ, is given by:

(8) $$\tau =\frac{Tr}{J},\;$$

where T is the applied torque, r is the radius of the cross-section, and J is the polar moment of inertia of the cross-section. Similar to Equation (5), the torsion is inversely proportional to J, which is again related to the fourth power of the radius, and thus, thin shell structures help prevent bending and torsion. The internal structure of the avian bone, depending on the forces experienced during the flight, can either have struts or ridges for reinforcement, as shown in Figure 12. The ridges in avian bone help to adjust to the torsional forces (T) experienced during the flight. Ridges are the protrusion of bones that lie flat against interior walls. They develop at a −45° angle to the horizontal axis to increase the structure’s resistance against the large tensile stress generated in torsion [48]. Ridges along this angle are most effective because, in torsion, axial stresses occur at 45 degrees to the longitudinal axis, as can be seen from Equations (9) and (10) [49].

(9) $$\sigma_{n}=-\tau \sin\left(2\theta \right),$$

(10) $$\tau_{nt}=-\tau \cos\left(2\theta \right),$$

where $\sigma_{n}$ is the normal stress, $\tau_{nt}$ is the shear stress, τ is the shear stress acting on the structure, and θ is the angle between the plane on which the stress components are resolved.

**Figure 13:**
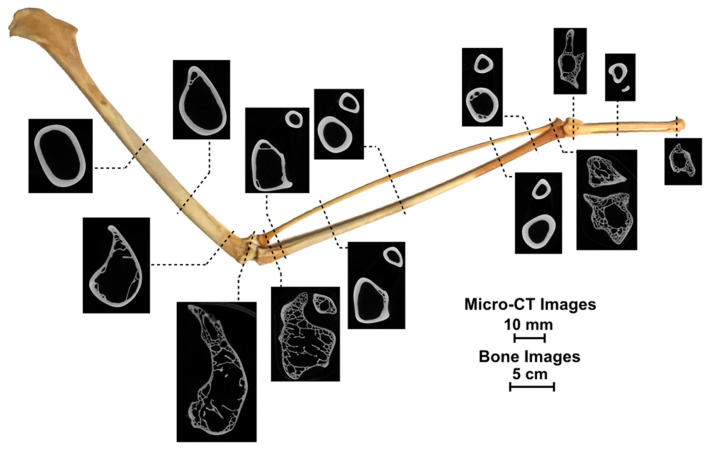
Micro-computed tomography (micro-CT) images revealing variations in density and thickness throughout the wingspan. Reproduced with permission from Ref. [50].

Struts are individual rods that span the interior of pneumatic bones. They are primarily located on the ventral side of wing bones in flying birds, where they appear to reinforce areas more susceptible to local buckling from combined bending and torsional forces [49]. Additionally, variations in density and wall thickness variations are likely to depend on the wing’s location due to spatial differences in bending and torsional moments, as illustrated in Figure 13 [50].

The important design parameters from BDT responsible for lightweight biological structures with the combined effect of bending and torsion resistance are summarized in Table 3. Similar to previously discussed case studies, a collective integration of dissimilar materials, porous features, and hierarchically optimized distribution of material delivers the desired functionality.

This section considered and presented various biological design architectures to achieve a specific functionality. Lightweight designs delivering high stiffness, improved impact resistance, and torsional/bending resistance were evaluated. The underlying scientific principles were analyzed and presented through the lens of BDT tools for each functionality. The following section discusses representative examples of commonly reported manufacturing/simulation approaches to realize bio-inspired designs. The overview of current methodologies is critically analyzed to identify potential gaps in the current methodology for future research opportunities.

## 4. Bio-Inspired Lightweight Structures for Impact Resistance: Manufacturing Case Studies

This section is specifically aimed at discussing representative examples of manufacturing and/or simulation research reports on bio-inspired designs to achieve impact resistance based on the biological examples discussed in Section 3.2, presented as a case study. Additional example case studies of manufacturing bio-inspired lightweight designs can be found in previous publications [51,52,53,54,55,56,57]. The design parameter(s) extracted from the biological examples, the manufacturing/simulation processes employed, and the resulting structural properties are discussed. The discussion provides a contextual summary of representative literature reports with adopted methodology and key results/conclusions. Through this discussion, current approaches in manufacturing bio-inspired lightweight functional designs are presented. At the same time, critical knowledge gaps and opportunities for utilizing BDT in progressing toward ‘manufacturing for design’ are highlighted.

**Figure 14:**
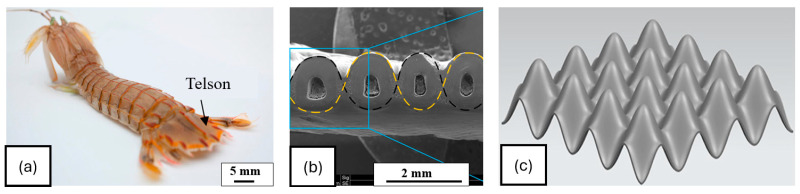
Bioinspired design of shrimp’s telson: (**a**) shrimp telson, (**b**) feature identification, (**c**) inspired design. Reproduced with permission from Ref. [58].

**Figure 15:**
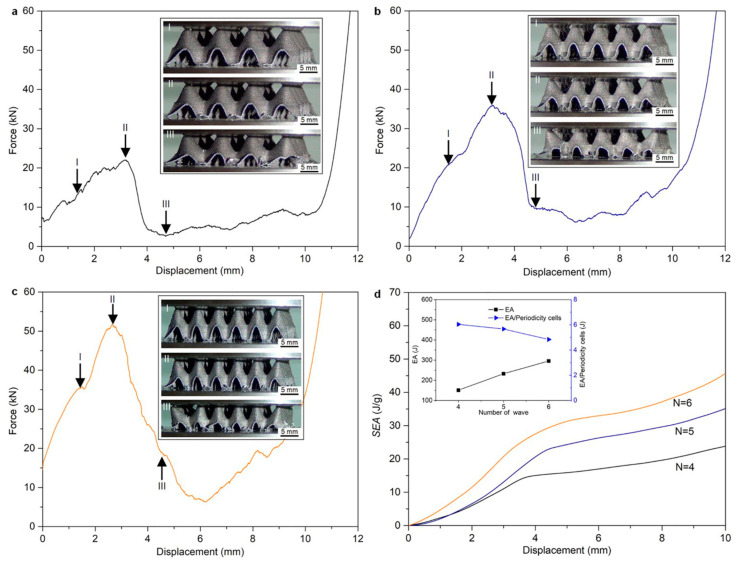
Mechanical performance of the DCP components processed through SLM during compression tests: (**a**) N = 4, (**b**) N = 5, and (**c**) N = 6. (**d**) Specific energy absorption (SEA)–displacement curve. Reproduced with permission from Ref. [58].

Yang et al. investigated the failure mechanisms of bio-inspired bi-directionally corrugated panel (DCP) structures under compressive loading inspired by the telson of the mantis shrimp, as represented in Figure 14 [41,58]. The study identified wave number (connected to the periodic distribution of a shape in BDT) and wall thickness (as a part of feature size in BDT) as the design parameters. The DCP structures, fabricated using selective laser melting (SLM) with AlSi10Mg powder, were examined for their compressive behaviors, stress distribution, deformation modes, and fracture mechanisms. The experimental variables were thickness: 0.3, 0.4, 0.5 mm and wave number: 4, 5, and 6, while the dimension of the structure was kept constant at 42 × 42 × 12.4 mm^3^. The force–displacement curves for several tested wave numbers for a constant thickness of 0.4 mm are depicted in Figure 15. The main findings indicate that increasing the wave number (N) resulted in more pronounced structural expansion effects, thereby altering the compressive force–displacement behavior and improving energy absorption efficiency. It is important to note that only the shape of the biological design was used for fabrication, while the size factor of the original biological structures was not utilized.

Additionally, the material properties of AlSi19Mg and its influence on other design parameters from BDT are not directly considered. In designing bioinspired structures, not all hierarchical design sizes can be manufactured due to limitations in manufacturability. For instance, micro- and nanoscale features are challenging to reproduce with conventional manufacturing processes at their respective length scales. However, additive manufacturing offers a solution to realize these intricate designs, although the precision and control over sub-micron features are typically limited.

Consequently, the design’s manufacturability becomes crucial in achieving bioinspired structures. The possibility of endless permutations and combinations of the design variables, in this case, thickness and wave number, makes it challenging to fabricate several design iterations to achieve an optimal design specific to a given material. Therefore, a solution is needed to determine whether a preliminary design space can be parameterized for manufacturability.

**Figure 16:**
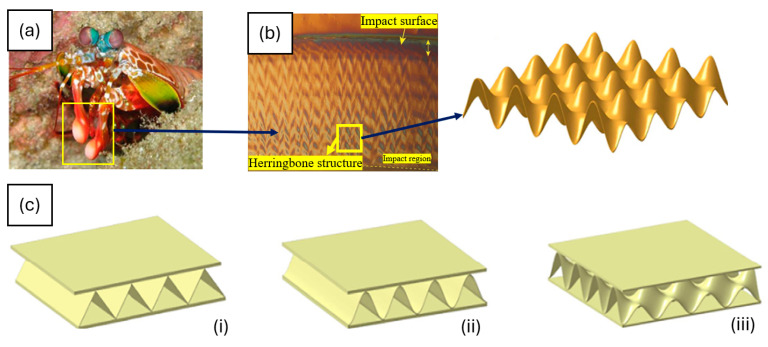
Bioinspired design of mantis shrimp’s dactyl heel: (**a**) peacock mantis shrimp dactyl heel, (**b**) feature identification and extraction, (**c**) designed structures comparing (**i**) triangular, (**ii**) single sinusoidal, and (**iii**) double sinusoidal sandwich structures [59].

In another approach, a double-sine corrugated (DSC) core sandwich structure inspired by the dactyl clubs of the peacock mantis shrimp was reported [59]. The DSC structures were compared with traditional triangular and single sinusoidal sandwich panels, as shown in Figure 16. Key factors such as wave amplitude, wave number, and core thickness (shape, size, and periodicity of features) were varied to optimize the design.

The research employed finite element analysis (FEA) to simulate quasi-static out-of-plane compression tests on these panels. The FEA results were validated experimentally for the triangular core structure. The force–displacement curve from the numerical model closely aligned with the test results, validating the accuracy of the simulation model. The results indicated that DSC structures significantly improved energy absorption and reduced initial peak force. The findings suggest that DSC sandwich panels, especially those with larger wave numbers and amplitudes, offer superior crashworthiness, making them promising for applications requiring high energy absorption and impact resistance.

Both the examples discussed above adopted wavy design features inspired by two distinct shrimp parts. However, the study using simulations was able to evaluate more design variables than the experimental study. This demonstrates that simulations could potentially assist in identifying a preliminary search space for bioinspired design architectures, providing insights into the parameters and their impact on the desired mechanical properties. However, experimental validation is often required to ensure the simulation’s accuracy and reliability and to confirm that the simulation model accurately represents the real-world system and its behavior under various conditions. Simulations often contain inherent assumptions and are simplified versions of actual experiments; therefore, they only provide approximate solutions to the modeled problem [60]. Due to its non-linear behavior, finding an analytical solution that links the design to its mechanical properties is often complex and challenging. However, it must also be noted that the exploration of design space through simulations should be coupled with the manufacturing perspective to ensure that the manufacturability aspect is considered.

**Figure 17:**
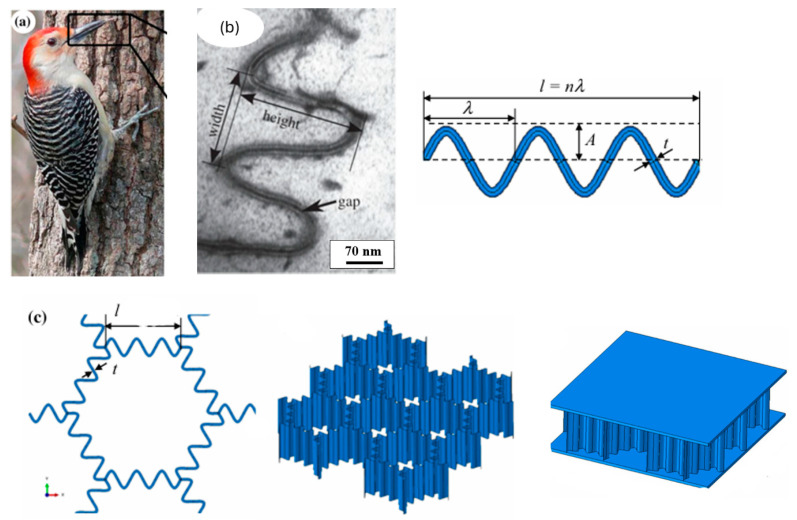
Bioinspired design of woodpecker’s (**a**) beak, (**b**) nanostructure and (**c**) the inspired design. Reproduced with permission from Ref. [15].

In another example, Ha et al. reported a novel bio-inspired honeycomb sandwich panel (BHSP) design based on the nanostructure of a woodpecker’s beak to improve energy absorption performance [15]. Unlike conventional honeycomb structures, the BHSP incorporates wavy cell walls. The authors used wave number and amplitude as the experimental design variables. The bioinspired design process, beginning from feature identification to design, can be seen in Figure 17. Using FEA, the researchers compared the energy absorption capabilities of the BHSP with conventional honeycomb sandwich panels (CHSPs). The results demonstrated that the BHSP exhibits superior energy absorption, with a specific energy absorption (SEA) increase of 125% and 63.7% compared to CHSPs of the same core thickness and volume, respectively. Parametric studies indicated that larger wave numbers and amplitudes in the BH core led to higher SEA.

Additionally, increasing the core thickness further enhanced SEA. While the biological analog provides design inspiration, the connection between biological design and engineering design remains at the BDT level only. The overall methodology of realizing a functional engineering design still highlights the need for optimization under the boundary conditions imposed by the material, manufacturing, and desired properties.

**Figure 18:**
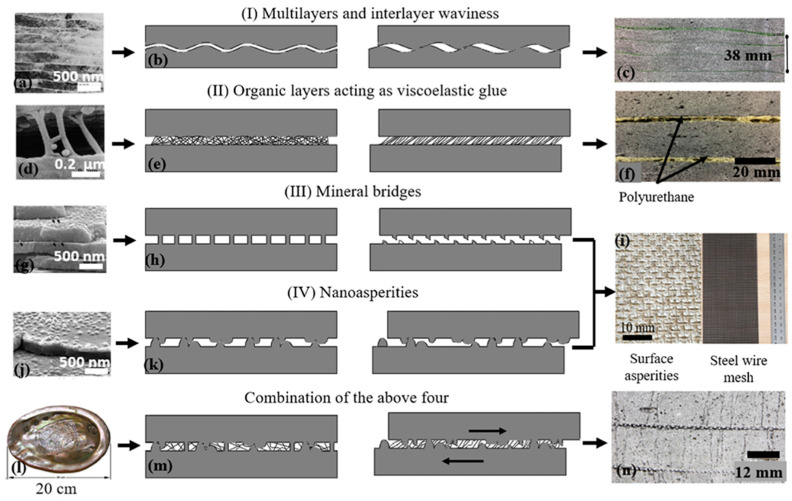
Examples of nacre-inspired architectures with different structures, each drawing inspiration from various aspects of nacre’s architecture depicting (**a**–**c**) multilayer and interlayer waviness, (**d**–**f**) organic matrix fibrils, (**g**–**i**) mineral bridges, (**i**–**k**) nano-asperities, and (**l**–**n**) combination of four mentioned features. Reproduced with permission from Ref. [61].

Another study investigated the flexural behavior and impact resistance of engineered cementitious composite (ECC) beams inspired by the microstructure of nacre [61]. The design parameters from the BDT used as experimental variables in the study include plate thickness and gradient density. Additionally, multi-material interfaces that incorporate different material chemistries were also reported. Figure 18 illustrates four tested beams: a monolithic beam, a layered beam with polyurethane (PU) interlayers, a layered beam with PU and surface asperities, and a layered beam with PU and steel wire mesh [61]. The results from quasi-static and low-velocity dynamic bending tests indicated that the bio-inspired beams exhibited significantly enhanced ductility, energy absorption, and impact resistance compared to the monolithic beams. Specifically, the layered beams with PU interlayers showed 150–300% more deformation and 55–195% higher energy absorption under quasi-static bending. Under dynamic bending, the beam with PU and steel wire mesh demonstrated the highest energy absorption, maintaining structural integrity even at high impact energies.

In a different study, Morsali et al. proposed a computational framework combining statistical and machine learning techniques with finite element analysis to create structure–property design strategies for brick-and-mortar composites inspired by nacre [62]. The framework involved testing twenty thousand designs with various feature combinations using finite element analysis to prepare the dataset. Subsequently, a decision tree machine learning model was created to delineate the optimal design space. Calculating the stress–strain responses for the 20,000 FEA models took approximately 50 h, whereas predicting the strength of one million geometries using the machine learning approach required only 6 s with the same computational resources and achieved comparable accuracy. While this highlighted the potential of machine learning algorithms in quickly evaluating the structural properties of many experimental variables, it does not fully couple with the capabilities of manufacturing processes from a systems-level perspective. Ideally, for truly utilizing the vast amount of biological design information for manufacturing engineering products, one must have clear interconnectivity between biological design information, science-driven and AI/ML-based methods/models, and manufacturing/material constraints.

**Figure 19:**
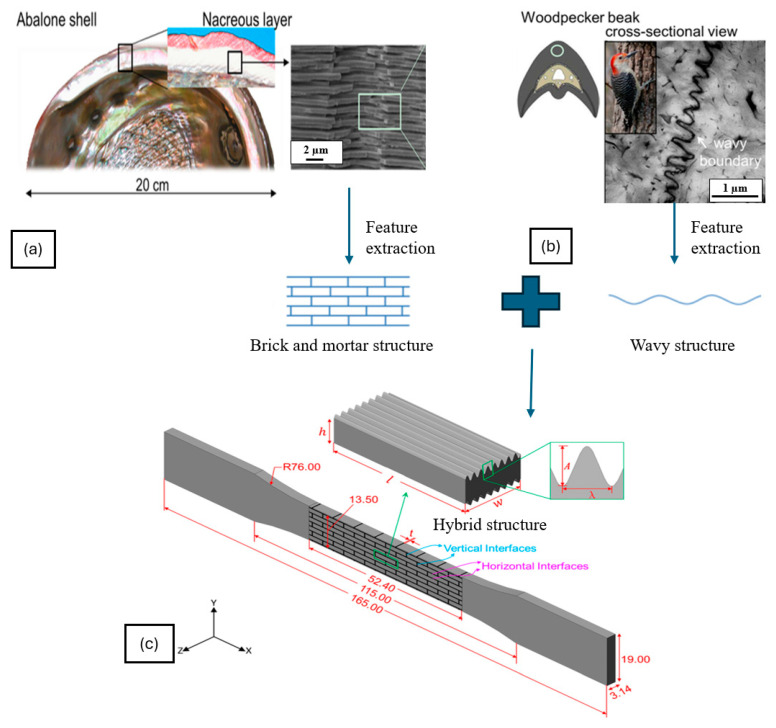
Hybrid bioinspired design inspired by nacre’s shell and woodpecker’s beak: (**a**) nacre’s shell feature extraction, (**b**) woodpecker’s beak feature extraction, (**c**) bioinspired hybrid structure. Reproduced with permission from Ref. [14].

Ding et al. presented a hybrid bio-inspired composite structure that integrates the brick-and-mortar 3D arrangement of nacre with the 2D wavy surface structure, such as those observed in woodpecker’s beak microstructure, to enhance energy absorption capability [14]. Figure 19 shows the hybrid bioinspired design. The research involves experimental and FEA methods to investigate the effects of tablet waviness and wave number on the mechanical properties of the composites. Stratasys J750 multi-material 3D printer was used to fabricate samples using photopolymers. The hybrid structure significantly improved strength, stiffness, and toughness compared to traditional nacre-like composites with flat tablets. Three failure modes were identified: soft phase failure and soft phase failure coupled with tablet and tablet break. The combination of soft phase failure and tablet break was crucial for achieving high toughness. FEA simulations revealed that stress concentration areas were primarily located at the peaks of the wavy surfaces. Increasing tablet waviness led to better load transfer and delayed fracture, enhancing energy absorption. The study concluded that an optimal balance between load transfer and stress concentration is essential for maximizing strength and toughness in bio-inspired composites. This demonstrated the feasibility of using a hybrid approach to effectively design lightweight bioinspired structures by integrating different design parameters from different species. The approach demonstrated the motivation to consider a vast library of lightweight biological designs as an information database that could be utilized to tune the appropriate designs suitable for an intended application within the constraints of the availability of materials and capabilities of the manufacturing operations.

To summarize this section, most studies, including those cited above, indicated that the design process often focuses on a single design parameter, referred to as a “feature”, of the biological species and thus potentially oversimplifying the complexity of biological systems. In reality, the relationship between the design and the resulting properties is influenced by numerous variables. Even when focusing on just one particular feature of a biological species, the interaction between various factors, such as material properties and manufacturing process, plays a crucial role in determining the final properties of the structure. In other words, the design may be sub-optimal because it fails to incorporate the multifaceted nature of biological systems, where multiple parameters and their interactions contribute to the overall functionality and performance. Hence, a holistic, system-level design methodology could further advance this field of manufacturing bio-inspired designs for a desired functional application, and a potential approach is proposed below.

## 5. Conclusions and Future Directions: Gaps and Opportunities

This paper provided an overview of various bio-inspired design parameters observed in biological examples for achieving lightweighting while maintaining desired functionality, emphasizing the underlying scientific principles. By investigating the anatomy and structural designs of different species in various environments, the study identified parameters that contributed to functional lightweighting. These design strategies are analyzed based on the parameters presented in BDT. Section 4 discussed representative examples of manufacturing and/or simulating bioinspired designs for lightweight applications with impact resistance properties to highlight currently employed bio-inspired design methodologies.

Reviewing various reports on bioinspired lightweight designs revealed that typical bioinspired design and manufacturing approaches include taking inspiration from a species of interest and abstracting the design parameters into practical manufacturable (material–design) combinations. Also, a significant portion of the current literature resorts to employing “trial-and-error” approaches when attempting to manufacture designs inspired by biological species with the desired functional properties and are not necessarily guided by the targeted engineering application constraints. This approach, while beneficial, needs a more holistic system-level view for coupling and decoupling the complexities between design (physical and chemical)–manufacturing process–product requirements for broader applicability. The key gaps and opportunities in the current bioinspired design methodologies are summarized below:

- Absence of scientific foundation: Many engineered bioinspired designs lack a fundamental understanding of the underlying scientific principles and rely heavily on directly replicating features without a deeper understanding of design–material–properties correlations.
- Non-modular design approach: The design process typically excludes BDT, which limits identifying the critical parameters responsible for achieving the desired functional property. This limitation often leads to evaluating multiple design variables in isolation without fully understanding how these variables interact with one another.
- Lack of initial design space identification: There is often no clear identification of the initial design space based on existing information or results, causing reliance on a “trial-and-error” approach for manufacturing bio-inspired designs.
- Limited insight from experimental studies and manufacturability: Existing reports and experimental studies might offer incomplete insights and suboptimal results due to limited design space exploration. This results in the limited availability of relevant data on the applicability of designs that can be produced using existing manufacturing processes, materials, and technologies. There is a gap in integrating simulations and machine learning models to expand and optimize the design search space and generate numerous digital test cases.
- Utilization of biological design toolbox: There is a potential gap in fully leveraging design parameters from various biological species or combining multiple parameters from a single species to enhance design outcomes.
- Limited input from manufacturing and production perspective: Similar to some of the above-identified gaps, the existing bio-inspired design strategies follow a linear approach to incorporate bio-inspired designs into an engineering application, seldom considering the design–material–manufacturing relationships. Availability of materials, manufacturability of identified designs, product considerations, and production-level constraints are thus not fully considered at the design stage.

**Figure 20:**
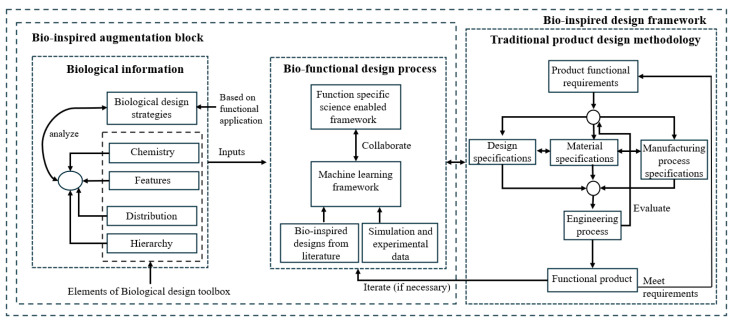
Proposed bio-inspired design framework for functional product fabrication.

To target the above gaps, the authors propose a data-informed, holistic design framework backed by fundamental science as a potential manufacturing design methodology to address the above challenges. The framework augments the traditional engineering product design methodology by adding bio-information and science-enabled functional design blocks, as shown in Figure 20.

The biological information block is based on proven biological designs for specific functionality, which in this study is lightweighting. It analyzes information from various biological species to identify design parameters such as chemistry, distribution, features, and hierarchy from BDT. This block utilizes analytical data from peer-reviewed literature and author analysis to produce quantitative data that support independent verification and validation. This process generates function-specific information necessary for the bio-functional design process block. Given the intricate design architecture of biological species, the biological information block may contain extensive information, some of which may not be relevant to the design or the target engineering application. The modularity provided by BDT serves as an outline for organizing and extracting relevant information. The bio-functional design process block combines the underlying scientific principles/mechanisms that enable a specific function of interest to create a science-driven design framework to identify the primary and secondary design parameters of interest. It also provides a collaboration link with a machine learning framework to enhance design and manufacturing processes, using data from bio-information blocks to guide the framework. The machine learning interface could use literature, experiments, and simulation data. These two blocks interface with the traditional functional product development process with the choice of design, materials, and manufacturing specifications guided by insights from the bio-functional design process. This interaction is critical from a functional product application standpoint where the engineering constraints, product specifications, availability and accessibility of suitable manufacturing process, scale-up, and cost considerations would provide a feedback mechanism to guide the framework for the desired product application. This systematic integration of data-driven insights from biology and literature with fundamental scientific principles within the bio-inspired design framework presents a comprehensive and robust approach.

In conclusion, this paper was focused on understanding the role of various design architectures found in biology to achieve lightweighting. Some of the commonly reported biological species that demonstrate lightweighting for specific functional responses were chosen as representative example cases. The various design strategies observed in these selected examples were decomposed into individual design tools, as represented through the ‘Biological Design Toolbox’ (BDT) for lightweighting. These individual tools/design elements (including chemistry, length scale hierarchy, building block features, and distribution) and their integration are guided by function and environment-specific boundary conditions. This was presented through the discussion of specific functions such as high stiffness, impact resistance, and torsional resistance with representative biological examples. At the end of each application, the design elements for the discussed biological examples are identified and connected to the BDT, and the fundamental science behind achieving the desired functional property is discussed. Current approaches to design, simulate, and manufacture bio-inspired functional engineering components were discussed from the perspective of BDT, illustrating how elements of the biological toolbox were utilized while highlighting gaps and opportunities in current methodologies. As highlighted in the paper, the current approaches rely on a trial-and-error methodology to incorporate bio-inspired designs into an engineering application, seldom considering the design–material–manufacturing relationships. The authors believe that the design elements in BDT can be viewed as ‘knobs’ or ‘levers’ that could be tuned by applying a science-enabled and machine learning approach to address the gap between biological designs and manufacturing functional structures based on the requirements and constraints of the target product application. While this review briefly introduced the novel bio-inspired design framework, its application to bio-inspired lightweight material manufacturing will be explored and validated in future research. This could offer a more robust and innovative pathway for developing optimized bioinspired designs.

## Figures and Tables

**Table 1:** Design parameters from BDT in lightweight, stiff biological examples.

Species	Chemistry	Feature	Distribution	Hierarchy
Bamboo	Cellulose, lignin	Fibrous Shape: Tubular Size: Diameter and wall thickness Aspect ratio: Diameter and height	2D Directionality: Radial Periodicity: Gradient density of fibers	Macro, micro
Horsetail	Silica, magnesium, potassium	Fibrous and porous Shape: Tubular Size: Diameter, wall thickness, ridge width, and cell wall thickness	2D and 3D Directionality: Radial Periodicity: Ridge angle, random cellular structure	Macro, micro
Plant of paradise	Cellulose	Fibrous and porous Shape: Tubular Size: Diameter, wall thickness, and cell wall thickness	2D and 3D Directionality: Radial and axial Periodicity: Random positioning of cells	Macro, micro

**Table 2:** Design parameters from BDT in lightweight impact-resistant biological examples.

Species	Chemistry	Feature	Distribution	Hierarchy
Beetle’s elytra	Chitin and protein	Porous Shape: Polygon Size: Edge length, wall thickness, and order of feature	2D Directionality: Axial fiber orientation Periodicity: Random	Micro
Mantis shrimp’ telson	Chitin and protein	Layered plates Shape: Sinusoidal Size: Wave number, amplitude, and wall thickness	2D Directionality: Lateral Periodicity: Wave number	Macro
Mantis shrimp’s dactyl heel	Chitin and protein	Fibrous and layered Shape: Cylindrical Size: Diameter Aspect ratio: Diameter to height	2D and 3D Directionality: Angular orientation of the fiber Periodicity: Layering	Macro, micro
Woodpecker’s beak	Chitin and protein	Plates Shape: Toothed plates Size: Waviness, width, height, discontinuity width and interlocking angles	2D Directionality: Lateral Periodicity: Tooth wave number	Macro, micro, nano
Nacre’s shell	Calcite and nacreous	Layered plates, viscoelastic Shape: Polygon plates Size: Length, width, height, biopolymer thickness	2D and 3D Directionality: Lateral and axial Periodicity: Random	Macro, micro, and nano

**Table 3:** Design parameters from BDT in lightweight torsional and bending-resistant biological examples.

Species	Chemistry	Feature	Distribution	Hierarchy
Avian skeleton	Collagen and calcium phosphate	Fibrous and porous Shape: Tubular Size: Diameter and wall thickness	2D and 3D Directionality: radial and axial Periodicity: Gradient density and thickness	Macro, Micro

## Data Availability

No new data were created during this review article. Please contact the authors for any questions regarding the collected and published information.
